# Signal integration by chloroplast phosphorylation networks: an update

**DOI:** 10.3389/fpls.2012.00256

**Published:** 2012-11-20

**Authors:** Anna Schönberg, Sacha Baginsky

**Affiliations:** Institut für Biochemie und Biotechnologie, Martin-Luther-Universität Halle-WittenbergHalle (Saale), Germany

**Keywords:** chloroplast, kinase, phosphatase, signal transduction, phosphorylation

## Abstract

Forty years after the initial discovery of light-dependent protein phosphorylation at the thylakoid membrane system, we are now beginning to understand the roles of chloroplast phosphorylation networks in their function to decode and mediate information on the metabolic status of the organelle to long-term adaptations in plastid and nuclear gene expression. With the help of genetics and functional genomics tools, chloroplast kinases and several hundred phosphoproteins were identified that now await detailed functional characterization. The regulation and the target protein spectrum of some kinases are understood, but this information is fragmentary with respect to kinase and target protein crosstalk in a changing environment. In this review, we will highlight the most recent advances in the field and discuss approaches that might lead to a comprehensive understanding of plastid signal integration by protein phosphorylation.

## CHLOROPLAST PROTEIN KINASES AND THEIR TARGETS: A SHORT OVERVIEW

Phosphorylation of thylakoid membrane proteins and its role in adjusting photosystem excitation pressure by “state transitions” was among the first reports on the function of protein phosphorylation in plants (Bennett, 1977; Allen et al., 1981). Surprisingly, the responsible protein kinases remained elusive because of technical constraints in their biochemical characterization, e.g., their low abundance, their membrane integration, and problems in the characterization of phosphorylation specificity in *in vitro* systems. With the help of genetics, the “state transition” kinase was first identified in *Chlamydomonas* and then – by homology – in *Arabidopsis thaliana*, and it was named Stt7 and STN7, respectively (Lemeille and Rochaix, 2010). At least two other protein kinases, STN8 and “thylakoid associated kinase 1 (TAK1)” that differ in function and target protein preference from STN7, are active at the thylakoid membrane system in Arabidopsis chloroplasts (Pesaresi et al., 2011). STN7 and potentially also STN8 are involved in short- and long-term adaptations of the photosynthetic machinery to changes in light quality and quantity (Bonardi et al., 2005; Bräutigam et al., 2009; Pesaresi et al., 2009). Long-term adaptations change the plastid metabolic state and are accompanied by a stable shift in the expression of genes for the two photosystems. It is therefore conceivable that the thylakoid-associated protein kinases are involved in signaling crosstalk between photosynthesis, the regulation of gene expression, and probably other metabolic functions.

The tailored adaptation of chloroplast functions to distinct photosynthetic states requires other chloroplast kinases as mediators that target specialized sets of proteins for regulation. Surprisingly, even systematic surveys only identified a small set of typical eukaryotic protein kinases in chloroplasts that alone cannot account for the signaling complexity implied by recent observations (Bräutigam et al., 2009; Pesaresi et al., 2009; Bayer et al., 2012). Instead, a group of atypical kinases of prokaryotic origin were recently identified, which comprise the two-component-like “chloroplast sensor kinase (CSK)” and a group of ABC1 kinases (Puthiyaveetil et al., 2008; Lundquist et al., 2012). While the chloroplast sensor kinase is well characterized, little is known about the function of the ABC1K group in organelles. Their ancestral function was in quinone biosynthesis and their name alludes to their critical role in the assembly of the “bc1 complex” (Lundquist et al., 2012). Most of the ABC1Ks in chloroplasts associate with plastoglobuli, where they are suspected to play a role in the synthesis of vitamin E and carotenoids (Vidi et al., 2006; Lundquist et al., 2012). However, functional proof for their role in the above syntheses or information on their target protein spectrum is currently missing.

While ABC1Ks and CSK are kinases of prokaryotic origin, there is increasing evidence that a typical eukaryotic second messenger is involved in phosphorylation-mediated chloroplast signaling, i.e., Ca^2^^+^. *In vitro*, several proteins are phosphorylated in a Ca^2^^+^-stimulated manner, among them the FtsH protease Var1, the Ca^2^^+^-sensing protein *Cas* and photosystem subunits (PsaN, PsbP-1, PsaH-2; Stael et al., 2012). The chloroplast concentration of free Ca^2^^+^ oscillates in response to different stimuli such as shift to darkness (Sai and Johnson, 2002), and different elicitors (Nomura et al., 2012; Stael et al., 2012). In such a dynamic system, Ca^2+^-dependent phosphorylation of chloroplast proteins would be a straightforward means to decode the Ca^2^^+^-signals. However, clear-cut evidence for Ca^2^^+^-dependent protein kinases in chloroplasts is missing. The “calcineurin B-like protein-interacting protein kinase 13 (CIPK13)” carries a functional N-terminal transit peptide that guides a truncated version of CIPK13 into chloroplasts (Schliebner et al., 2008; Bayer et al., 2012). However, localization experiments with the full-length protein tagged with GFP at its C-terminus contradict the chloroplast localization data (own unpublished data). Furthermore, CIPK13 requires a calcineurin B-like protein (CBL) for catalytic activity, which was not identified in chloroplasts so far. Therefore, the search for Ca^2^^+^-regulated protein kinases in chloroplasts is ongoing.

Despite the relatively small number of established chloroplast protein kinases, their substrate spectrum and their regulation are only partially understood. Systematic phosphoproteome surveys identified around 300 phosphoproteins and 900 phosphopeptides (Durek et al., 2010). A comprehensive understanding of chloroplast signal integration requires that the *in vivo* targets of chloroplast kinases and the conditions for their phosphorylation are known. With the kinases and the phosphoproteins at hand, large-scale, unbiased target protein surveys by comparative quantitative phosphoproteomics are now feasible (Reiland et al., 2011; Ingelsson and Vener, 2012). Furthermore, peptide chip technology enables the analysis of phosphorylation activity on several hundred substrates in parallel (Thiele et al., 2009). Both large-scale methods offer the type of unbiased information that is necessary for the characterization of a network with unknown connections. In this review, we will highlight the most recent advances in phosphoprotein network characterization, focusing on the STN7/STN8 kinases, CSK and the plastid transcription kinase “casein kinase II (pCKII),” and on two recently identified protein phosphatases that counteract STN7/STN8 activities (Pribil et al., 2010; Shapiguzov et al., 2010; Samol et al., 2012). With our selection we can only cover a small part of this fascinating field and refer the reader to the numerous excellent recent reviews on chloroplast protein phosphorylation for further information (Pesaresi et al., 2011; Rochaix, 2011; Puthiyaveetil et al., 2012; Tikkanen and Aro, 2012).

## AN EMERGING ROLE FOR PROTEIN KINASES IN THE REDOX CONTROL OF PLASTID AND NUCLEAR GENE EXPRESSION

**FIGURE 1:**
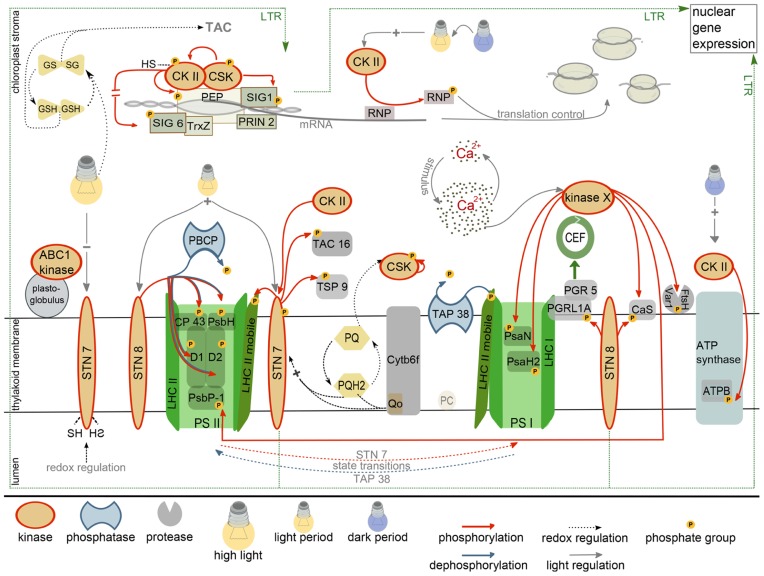
**Current state of research on the chloroplast phosphorylation network**. Depicted are established kinase regulations together with the known kinase targets (red, red arrows). Phosphatases that counteract kinase activity are presented in blue. The regulation of the long-term-response (LTR) by kinases of the thylakoid membrane system involves changes in nuclear gene expression and is marked with a dashed line. For further details see main text. CEF, cyclic electron flow; GSH, GSSG, reduced, oxidized glutathione; TAC, transcriptionally active chromosome; PEP, plastid encoded RNA polymerase; CKII, casein kinase II; CSK, chloroplast sensor kinase; RNP, RNA-binding protein; PQ, PQH_2_, plastoquinone, plastoquinol; PSII, PSI, photosystem II/I; ATPB, β subunit of ATP synthase; PC, plastocyanin; SIG1, SIG6, sigma factor 1/6; TrxZ, thioredoxin; PRIN2, plastid redox insensitive; LHC, light harvesting complex.

Plastid protein kinases have a role in controlling short (STR) and long-term responses (LTR) of the two photosystems and the metabolic state of the chloroplast to changing light conditions. Both responses are controlled by the functional status of the photosynthetic electron transport chain, in part by means of reduction/oxidation (redox) properties of redox sensors. At least the LTR is accompanied by changes in plastid and nuclear gene expression that result in the stable adaptation of plastid metabolism to environmental conditions. Redox regulation of nuclear and plastid gene expression is channeled through the plastid transcription system. For example, the “plastid redox insensitive 2 (*prin2*)” mutant is unable to initiate the known redox-induced changes in the expression of nuclear encoded light-harvesting complex (LHC) proteins. PRIN2 associates with the plastid encoded RNA polymerase (PEP) complex, and its absence affects plastid transcription in a way that is characteristic for a defect in the plastid transcription machinery (Pfalz et al., 2006; Kindgren et al., 2012). Thus, PRIN2 – as part of the plastid encoded RNA polymerase – generates a retrograde signal that connects the redox information from the photosynthetic electron transport with the expression of plastid and nucleus encoded genes (**Figure 1**; Fey et al., 2005; Pfannschmidt and Yang, 2012; Kindgren et al., 2012). The mechanism of perception and transduction of the redox signals are currently unknown.

Plants that lack the light-regulated STN7 kinase are affected in the LTR, arguing for its crucial function in the acclimation response (Bräutigam et al., 2009). The STN7 kinase is regulated by light and therefore could serve as redox sensor that either directly phosphorylates components of the plastid metabolic and/or gene expression system, or that transduces the signals by means of a phosphorylation cascade. Activation of STN7 depends on the reduction of the plastoquinone pool and the binding of reduced plastoquinone to the quinol(Q_p_)-binding site of the cytochrome b_6_/f complex (Rochaix, 2011). The regulation of STN7 also involves plastoquinol-independent redox signals because STN7 is inactivated under high-light by reduction of a disulfide bridge in the thylakoid lumen. STN7 inactivation is mediated by the ferredoxin/thioredoxin system (Rintamaki et al., 2000; Lemeille et al., 2009), thus, the redox signals must be relayed across the thylakoid membrane. Interestingly, STN7 is not only a passive redox sensor, but also maintains the redox properties of the electron transport chain under rapidly fluctuating light intensities. This occurs indirectly by cooperative effects of LHCII phosphorylation, NPQ and PGR5-dependent control of electron flow on the stability and functionality of PSI (Grieco et al., 2012).

Several analyses were conducted to identify the target proteins of STN7 and STN8 to understand the connection between photosynthetic performance and the control of gene expression. STN7 was originally identified as the “state transition” kinase that phosphorylates proteins of the light harvesting complex II (LHCII; Rochaix, 2011). Comparative phosphoproteomics between wild-type and *stn7* identified furthermore TSP9 (At3g47070) and pTAC16 (At3g46780) as new potential STN7 targets. TAC16 is a subunit of the “transcriptionally active chromosome,” a high molecular mass complex comprising the PEP polymerase, and as such a potential mediator between STN7 phosphorylation activity and the regulation of plastid transcription. However, TAC16 is not a core component of the plastid transcription machinery and it is distributed between the TAC and thylakoid membranes (Ingelsson and Vener, 2012). Furthermore, TAC16 accumulates differently from most other TAC subunits and it is assumed that it anchors the plastid RNA polymerase at the thylakoid membrane (Majeran et al., 2012; Motohashi et al., 2012). The exact quantitative distribution of TAC16 between the transcription complex (TAC) and the thylakoid membrane may be regulated by STN7-dependent phosphorylation, but an influence of TAC16 phosphorylation on plastid transcription is currently not known (**Figure 1**; Ingelsson and Vener, 2012).

STN8 is also activated by light via a reduced plastoquinone pool, but in contrast to STN7, STN8 is also active under high-light conditions (**Figure 1**; for details see, e.g., Rochaix, 2011; Pfannschmidt and Yang, 2012; Puthiyaveetil et al., 2012). STN8 was originally identified as the kinase responsible for phosphorylation of the core subunits of PSII, e.g., CP43, D1, D2, and PSBH and the Ca^2^^+^-sensing protein *Cas *(Bonardi et al., 2005; Vainonen et al., 2005, 2008). Light-dependent phosphorylation of *Cas* may connect STN8 with Ca^2^^+^-signaling. In Chlamydomonas, *Cas* controls high-light induced changes in gene expression and *Cas* knock-down lines are light sensitive with impaired activity and recovery of PSII (Petroutsos et al., 2011). In higher plants *Cas* is required for signal-induced stomata closure and is dual targeted to chloroplasts and mitochondria (Nomura et al., 2008; Carrie et al., 2009). Despite its numerous signaling functions, *cas* mutants have a surprisingly mild phenotype with a slight growth retardation but with unaffected photochemical properties (Vainonen et al., 2008). Large-scale quantitative phosphoproteome profiling revealed several other potential STN8 targets, all of which are associated with the plastid thylakoid or inner envelope membrane system. One of the STN8 targets is PGRL1 that associates with PGR5 and such controls the switch from linear to cyclic electron flow (DalCorso et al., 2008; Reiland et al., 2011; Ingelsson and Vener, 2012). Functional analyses revealed a slowing down of the transition from linear to cyclic electron flow in *stn8* mutants that is similar to that observed with the *pgr5* mutant. This suggests that STN8 influences cyclic electron flow potentially by phosphorylation of the PGRL1/PGR5 complex (**Figure 1**; DalCorso et al., 2008; Reiland et al., 2011).

The recent identification of a redox-regulated protein kinase that resembles two-component sensor kinases from prokaryotic systems opened up a new perspective on photosynthetic signaling. The CSK may represent the postulated redox sensor that directly regulates plastid transcription via phosphorylation, as predicted in the CoRR hypothesis ( Co-localization for Redox Regulation; Allen, 1992). CSK knockout mutants are unable to repress the transcription of PSI subunits for photosystem stoichiometry adjustment under conditions that favor PSI excitation (PSI light; Puthiyaveetil et al., 2008). Under these conditions, transcription of PSI subunits is repressed in wild-type to adjust PSII and PSI excitation pressure. Interestingly, oxidized plastoquinone activates auto-phosphorylation of CSK, potentially at a tyrosine residue. Thus, both redox states of the plastoquinone pool, reduced and oxidized, comprise information that is translated into regulatory phosphorylations by two different kinases, CSK and STN7 (**Figure 1**).

Chloroplast sensor kinase, as a reminiscent two-component sensor kinase is expected to operate in conjunction with a response regulator. However, because CSK lacks the histidine residue required for sensor kinase activation, the signal transduction chain may differ from the common sensor kinase/response regulator-type that is prevailing in prokaryotes. In search for response regulator proteins that interact with CSK, two-hybrid assays were performed and CSK was found to interact with two proteins involved in transcriptional regulation, “sigma factor 1 (SIG1)” and the plastid transcription kinase cpCKII (Puthiyaveetil et al., 2012). Based on the interaction data it was suggested that CSK may directly control plastid transcription by SIG1 phosphorylation. The interaction with cpCKII could constitute a redox-regulated *regulon* that controls plastid transcription activity. Plastid CKII is a pleiotropic kinase that phosphorylates numerous target proteins among them RNA-binding proteins (RNPs), components of the PEP complex, and sigma factors (Reiland et al., 2009). Phosphorylation of the PEP complex and the sigma factors results in an unspecific repression of transcription activity (Baginsky et al., 1999; Baena-Gonzalez et al., 2001). Notably, cpCKII itself is inactivated by reduced glutathione such connecting chloroplast redox homeostasis with the regulation of transcription (Baginsky et al., 1999). In a recent paper, Link and colleagues confirmed direct redox control of cpCKII activity and identified regulatory SH groups that are crucial for SIG6 phosphorylation *in vitro *(Turkeri et al., 2012). Two out of four conserved cysteine residues in Arabidopsis cpCKII, i.e., Cys158 and Cys313, are essential for catalytic activity. Oxidation by diamide further revealed dimerization of cpCKII that depends on a disulfide bridge involving Cys182. Oxidation and dimerization both result in the inactivation of kinase activity (**Figure 1**).

This SH-group control of cpCKII activity and its interaction with CSK and SIG1 suggests that CSK and cpCKII form a regulatory unit that controls plastid transcription. The specificity of cpCKII-mediated transcriptional regulation could be exerted by its interaction with CSK, SIG1 or the polymerase complex directly, while cpCKII not associated with the transcription system may be responsible for the regulation of other chloroplast functions, e.g., energy metabolism and posttranscriptional processes of gene expression (Baginsky et al., 1997; Reiland et al., 2009). Because cpCKII is under phosphorylation control (Baginsky et al., 1999). CSK regulation of cpCKII catalytic activity by phosphorylation could balance the need for specific and non-specific regulation of transcription by phosphorylation. The following scenario put forward by Allen and colleagues (Puthiyaveetil et al., 2012) elegantly summarizes the data on interaction and regulation of the *regulon* components: overexcitation of PSI is sensed as oxidized plastoquinone pool that activates CSK resulting in SIG1 phosphorylation and down-regulation of transcription of PSI subunits (as revealed by *in vitro* transcription assays; Tiller and Link, 1993; Baginsky et al., 1999; Baena-Gonzalez et al., 2001). Under low-light, cpCKII is unphosphorylated and active and such acts as unspecific transcriptional repressor by phosphorylation of sigma factors and PEP subunits (see above). In order to allow photosystem stoichiometry adjustment, the general block on transcription is removed by inactivation of cpCKII through CSK phosphorylation and replaced by a specific block that affects SIG1 controlled genes (**Figure 1**). Although phosphorylation control of cpCKII activity was only observed *in vitro* with “protein kinase A (PKA)” from bovine heart, this model could explain the specificity of CKII-mediated regulation of chloroplast transcription. It should be noted, however, that the suggested phosphorylation events in this regulatory system are just anticipated and biochemical proof for any of these *in vivo* is missing. An excellent review with a detailed discussion is available from Allen and colleagues (Puthiyaveetil et al., 2012).

The large number of cpCKII targets requires fine-tuned control of kinase activity. In fact, some regulatory functions of cpCKII are mutually exclusive. For example, there is evidence for light-induced phosphorylation of RNPs during etioplast to chloroplast conversion (Kleffmann et al., 2007). Phosphorylation of RNPs weakens their interaction with plastid mRNA, thus releasing a translational block under conditions when higher translation rates of photosynthetic proteins are needed (Loza-Tavera et al., 2006). The phosphorylation site and the properties of the protein kinase responsible for RNP phosphorylation strongly hint at cpCKII (Kanekatsu et al., 1993; Reiland et al., 2009). Thus, cpCKII must be activated by light. On the other hand, cpCKII also phosphorylates the β-subunit of the ATPase complex (Kanekatsu et al., 1998) preferentially at the end-of-night, i.e., in the dark, probably to avoid ATP hydrolysis under conditions when electron transport does not occur (Reiland et al., 2009). Thus, cpCKII specificity and activity must be tightly controlled, potentially by specific interaction of cpCKII subpopulations with different protein complexes (**Figure 1**).

Casein kinase II is not only under phosphorylation control itself; it may also phosphorylate other chloroplast kinases. A potential target kinase for cpCKII phosphorylation is the state transition kinase STN7, which is phosphorylated at C-terminal threonine residues that resemble CKII target motifs (**Figure 1**; Reiland et al., 2009). While such a crosstalk could serve as a platform for signal integration, functional analyses showed that phosphorylation of STN7 does not affect its catalytic activity or its regulatory properties, but rather its stability (Willig et al., 2011). The STN7 kinase in Arabidopsis and its ortholog Stt7 in *Chlamydomonas* are both destabilized under prolonged “state 1” conditions, i.e., when phosphorylation of the LHC proteins does not occur. Under these conditions, phospho-mimic mutants in which the phosphorylated threonines are exchanged with aspartate are stabilized compared to their wild-type counterpart suggesting that the negative charge at the C-terminus of the kinase may protect it from degradation (Willig et al., 2011). STN7 kinase turnover may be regulated by the many chloroplast proteases that are involved in chloroplast protein homeostasis, e.g., Clp, Deg, and/or FtsH (Adam et al., 2006). However, the *in vivo* significance of phosphorylation-dependent STN7 stabilization remains unclear because in several protease-, phosphatase-, and kinase mutants (note that a cpCKII mutant was not tested) STN7 stability was unchanged compared to wild-type (Willig et al., 2011). Nonetheless, the above data highlight another important mechanism that contributes to chloroplast signaling: protein degradation by the complex protease network.

## DEPHOSPHORYLATION COUNTERACTS THE ACTIVITY OF THYLAKOID ASSOCIATED KINASES

Regulatory systems that operate via protein phosphorylation must comprise activities that turn off phosphorylation-induced signals in response to changing conditions. In principle there are two possibilities to turn off phosphorylation-triggered signals: (i) the irreversible phosphoprotein degradation by proteases and (ii) the reversible release of the phosphate group by phosphatases. Early reports showed that different phosphatase activities with distinct kinetic properties act on thylakoid phosphoproteins. LHCII proteins are most rapidly dephosphorylated, followed by D1 and D2, and then by CP43 and PsbH (Silverstein et al., 1993). Consistent with the kinetic data, two different protein phosphatases were recently identified that specifically counteract phosphorylation activity of STN7 and STN8, respectively. The PPH1/TAP38 protein phosphatase was identified in a genetic screen for mutants with a defect in protein dephosphorylation upon “state 2” to “state 1” transition. This phosphatase localizes to the stroma lamellae of thylakoid membranes (**Figure 1**). Phosphatase-deficient plants remain longer in “state 2” compared to wild-type. Biochemical analyses showed that the phosphorylation status of LHC complex proteins correlates inversely with the abundance of the PPH1 gene product. Together, these data suggest that PPH1 specifically counteracts the activity of STN7 (Pribil et al., 2010; Shapiguzov et al., 2010). Surprisingly, there is almost no co-expression between PPH1 and STN7 in gene expression networks such as ATTED II (Obayashi et al., 2009), suggesting that LHCII dephosphorylation is probably not the only function of PPH1.

This situation is different for the “PSII core phosphatase (PBCP)” and its counteracting kinase STN8 (**Figure 1**). PBCP dephosphorylates PSII core proteins and is involved in thylakoid stacking. Other targets of STN8 such as the Ca^2^^+^ sensing protein *Cas* and PGRL1 were either not affected in the mutant or not analyzed (Samol et al., 2012). There is some overlap in the function of PPH1 and PBCP, because plants that overexpress PBCP are affected in state transitions and also show slightly altered LHCII phosphorylation kinetics (Samol et al., 2012). The ATTED II co-expression network of PBCP comprises STN8, SIG3, and CSK in close proximity, which could suggest that PBCP not only counteracts STN8 activity but also has a role in the dephosphorylation of the PEP complex and/or sigma factors. As detailed above, such a PEP phosphatase is required to counteract the activities of CSK and cpCKII and to release the phosphorylation-induced block of transcription.

## CONCLUSIONS AND OUTLOOK ON NEW APPROACHES

With the players and the functional genomics tools at hand, we are in a position to characterize the chloroplast protein phosphorylation network in greater detail. Of particular interest are the nodes for crosstalk between different chloroplast functions, especially the connections between photosynthesis and the regulation of chloroplast and nuclear gene expression. Analytical approaches to map such higher-order network connections require unbiased assays of kinase activity on unknown targets *in vitro* and approaches to determine changes in protein phosphorylation states *in vivo*. The *in vitro* characterization of kinase-target protein specificity can be performed on peptides or proteins in a multi-parallel *in vitro* assay. In the “kinase client assay (KiC)”, cocktails of synthetic peptides are used to measure phosphorylation activity of a purified kinase. Following the phosphorylation reaction, the peptide mixture is analyzed by mass spectrometry and phosphorylated peptides are identified and quantified by spectral counting or ion intensity measurements. A proof-of-concept study using a mixture of 79 peptides (11–20 mers) and purified pyruvate dehydrogenase kinase (PDK) showed a surprisingly high specificity of this assay system (Huang et al., 2010). Similar to the above, but with solid-phase immobilized peptides on a glass slide or a membrane, “Peptide Chips” allow the analysis of phosphorylation activity of a purified kinase or a protein extract *in vitro *(Thiele et al., 2009). The advantages of “Peptide Chips” are their high peptide density (more than 1000 peptides on one chip), while disadvantages are unspecific surface effects on kinase activity that result from the peptide immobilization and the spacing between the target amino acid and the solid phase. With “Protein Arrays,” kinase activity is measured *in vitro* with several protein substrates in parallel, either in solution or immobilized on a membrane. This assay represents a standard kinase assay on selected substrates, in which the transfer of radioactive phosphate from γ-^32/33^P-ATP or GTP onto target proteins is measured and quantified. Protein arrays are available for Arabidopsis and were used for the identification of MAP kinase targets (Feilner et al., 2005; Popescu et al., 2009).

The* in vivo* characterization of phosphorylation quantifies the phosphorylation state of putative kinase client proteins in wild-type compared to a kinase mutant, or under different environmental conditions. A lower phosphorylation state of a protein in the kinase mutant suggests a direct or indirect kinase-target protein relationship. The method of choice for this approach is the quantitative comparative phosphoproteome analysis by mass spectrometry, because it does not necessitate a hypothesis on target proteins, i.e., it is unbiased (Reiland et al., 2011; Ingelsson and Vener, 2012). Such a comparison requires a method for relative phosphopeptide quantification. Reiland et al. (2011) reported a label-free approach that uses the measured phosphopeptide intensity between liquid chromatography (LC) runs with wild-type and the *stn8* mutant (the so called “extracted ion chromatograms”) for relative quantification. Because phosphopeptide elution times may be shifted between different LC runs, this method requires an algorithm for chromatographic alignment (reviewed in Baginsky, 2009). Labeling approaches using stable isotopes are less error prone because they allow mixing samples to perform only one LC run for comparative analyses. The relative quantification is done by comparing the extracted ion chromatogram of the peptide containing the heavy isotope with that of the peptide containing the light isotope. ^14^N/^15^N labeling was used to study the abscisic acid (ABA)-dependent dynamic changes in the phosphorylation network (Kline et al., 2010) and the changes in protein phosphorylation after nitrogen starvation and resupply (Engelsberger and Schulze, 2012). These tools are now available for the characterization of chloroplast phosphorylation networks but further improvement is needed to assess subtle changes in phosphorylation stoichiometry of regulator proteins. Nonetheless, forty years after the initial reports, research on chloroplast protein phosphorylation has regained its original momentum.

## Conflict of Interest Statement

The authors declare that the research was conducted in the absence of any commercial or financial relationships that could be construed as a potential conflict of interest.
